# Perceived Democraticness of Parties From Citizens’ Perspectives: Evidence From Canada

**DOI:** 10.1177/00323217251324025

**Published:** 2025-03-15

**Authors:** Ka Ming Chan, Laura B Stephenson

**Affiliations:** 1 School of Geography, Politics and Sociology, Newcastle University, Newcastle upon Tyne, UK; 2 Department of Political Science, University of Western Ontario, London, ON, Canada; 3 The Consortium on Electoral Democracy (C-Dem), London, ON, Canada; 4 Centre for the Study of Political Behaviour, University of Western Ontario, London, ON, Canada

**Keywords:** political parties, democratic norms, citizen perceptions, motivated reasoning, Canada

## Abstract

Experts consider some parties to be democratic and others undemocratic, based on criteria related to political pluralism. But we know little about how citizens perceive a party’s commitment to democratic norms and whether these perceptions are vulnerable to change. Building upon the motivated reasoning literature, we answer these questions with data from a nationally representative sample in Canada – a well-regarded liberal democracy. First, our descriptive findings show that voters uniformly engage in motivated responding, perceiving their in-party as more committed to democratic norms than the out-party(-ies). Second, leveraging the 2022 Canadian trucker convoy, we prime respondents about the mainstream parties’ undemocratic behaviours (according to scholarly standards). Our experiment demonstrates asymmetrical information updating that supports motivated reasoning: voters maintain their in-party perceptions but they update perceptions of out-parties to be more undemocratic if they hold strong opinions about the convoy. We discuss how these findings enrich the democratic recession literature.

## Introduction

Evaluating whether political parties are democratic or undemocratic is a common exercise for academics. A prominent example is the V-Party data, which asks scholars to evaluate to what extent a party is committed to democratic norms (Düpont et al., 2022; Medzihorsky and Lindberg, 2024). The evaluation criteria include whether a party demonizes its opponents, adheres to free and fair elections, violates minority rights and encourages the use of violence against opponents. Considering the United States, the change in the Republican Party’s rating fits our scholarly intuition: from 2014 to 2018, its anti-pluralism index – a summary index of the above criteria – moved in the undemocratic direction by 0.236 units along a 0–1 scale. Another example is the Global Party Survey (GPS): its 2019 questionnaire asks country experts ‘Where do parties currently stand on liberal democratic principles, norms and practices?’ (Norris, 2020). Again, this dataset concurs with our understanding of what is considered democratic. In Germany, all parties are considered to respect liberal democratic principles and norms, except for the AfD – a far-right party affiliated with extremist movements.^1^ Note that these expert opinion datasets are not coded by a single scholar in each country, but rather by multiple scholars to provide a reasonable estimate of experts’ evaluations. Hence, these expert opinion indices indicate how scholars judge the democratic quality of a party.

Yet, politicians and parties often frame ‘democracy’ and ‘democratic behaviour’ much differently than experts. Instead of applying the above criteria, it is common to hear claims and accusations related to democracy made in rhetorical, inflammatory and often untrue ways. For instance, after Trump lost the 2020 election, he asserted the Democratic Party stole the election, while the Republicans were protecting the ‘democratic process’ (Brown, 2020). Another illustration comes from Canada, a well-regarded liberal democracy. In 2008, after the opposition parties agreed to form a coalition government if the minority Conservative government lost a confidence vote, the Conservatives quickly labelled the coalition ‘undemocratic’ (*CBC News*, 2008). This labelling process is puzzling, for scholars would hardly judge inter-party agreements as violating democratic norms. Nevertheless, these examples suggest two things. First, ‘democracy’ is a label used by politicians to their advantage: they label the in-group as democratic and the out-group as undemocratic. Second, the ‘democracy’ label used by politicians can be rather detached from the substantive criteria that concern scholars. Considering the vast literature that shows the impact of party cues on citizens’ opinions (Arceneaux and Truex, 2023; Druckman et al., 2013; Nicholson, 2012), one can expect the perception of whether a party respects democratic norms might also be affected by party cues. However, we know little about how citizens perceive the democraticness of different parties and whether these perceptions are vulnerable to change. This is an important consideration in an era of democratic backsliding.

To fill this gap, our research builds upon the motivated reasoning literature and uses a nationally representative survey in Canada to explore the democraticness of different parties from citizens’ perspectives. We suggest the Canadian case is a crucial one because both the GPS and the V-Party index consider all mainstream parties to be committed to democratic norms.^2^ If expert opinion reflects citizens’ perceptions, we should not see partisan bias in perceptions of whether a party respects democratic norms, and political messages should hardly change such perceptions. Our findings suggest otherwise. First, our descriptive results show that voters uniformly engage in motivated responding: they perceive their in-party as more committed to democratic norms than the out-party(-ies). Second, through a pre-registered experiment that leverages the 2022 Canadian trucker convoy, we provide respondents with a prime about party behaviours that would be judged undemocratic by scholarly standards.^3^ In line with the motivated reasoning literature, we uncover an asymmetrical information-updating process between in-party and out-party supporters. Overall, our findings suggest that there is motivated responding when it comes to citizens’ perceived democraticness of a party, and this perception is vulnerable to change by political messages.

Our research contributes to works on citizens’ perceptions of parties, motivated reasoning and democratic backsliding. Recent studies bridge the latter two strands of literature by showing that citizens engage in motivated responding in terms of conceptualizations of democracy (Bryan, 2023), perceived quality of democracy in their country (Carey et al., 2019; Flavin and Shufeldt, 2024) and politicians’ undemocratic behaviour (Krishnarajan, 2022). We extend this research agenda by directly asking about *citizens’ perceptions of whether different parties respect democratic norms and practices*. We see our research closest to the work by Pasek et al. (2022): their correlational analysis illustrates that US citizens perceive that their in-party Congressperson respects democratic norms and practices more than the out-party Congressperson. Yet, our research departs from theirs in two regards. First, we analyse a multi-party system where the in-party and out-party(-ies) dynamic is more nuanced; second, we use a pre-registered experiment to investigate whether political messages can change these perceptions.

## Democracy as a Label and Perceived Democraticness of Parties

In the recent literature on democratic backsliding, various researchers suggest that politicians and parties use ‘democracy’ as a label that is detached from the substantive criteria applied by experts. For instance, Lührmann (2021) argues that parties regarded by experts as anti-pluralist, such as the United Socialist Party of Venezuela, the AKP in Turkey and the Fidesz Party in Hungary, often claim they stand for ‘true democracy’. Likewise, a recent case study of Israel suggests the right-wing governing party Likud, which is rated as undermining democratic norms and practices in the GPS dataset,^4^ adopts ‘democratic rhetoric’ to legitimize its authoritarian policies that restrict minority rights (Perelman, 2024). On the other hand, when the opposition camp tried to stall the executive aggrandizement of Mexican President Andrés Manuel López Obrador, he denounced the opposition as aspiring to restore the ‘authoritarian’ ancient regime (Schedler, 2023), even though his party Morena is rated by V-Party as the most anti-pluralist in Mexico. From these examples, we can see there is a gap between expert opinion and the labelling process undertaken by political elites. That is, what scholars conventionally regard as an authoritarian party can be portrayed as ‘democratic’ by politicians, and vice versa. Given the various studies that show the impact of party cues on public opinion (Arceneaux and Truex, 2023; Druckman et al., 2013; Nicholson, 2012), we suggest this labelling process should affect citizens’ perceptions of whether different parties respect democratic norms and should lead to motivated responses.

Following Collier et al. (2006) and De Jonge (2016), we posit that the ‘democratic’ label is a desirable attribute while the ‘undemocratic’ label is an undesirable one. We consider this assumption plausible because, from the above examples, even conventional authoritarians often frame themselves as ‘democratic’ (Chapman et al., 2023; Lührmann, 2021; Schedler, 2023). Along this line of thought, other studies also highlight that conventional authoritarians and military coup leaders adopt the rhetoric of ‘democracy’ to satisfy global norms (Gorman, 2016; Yukawa et al., 2020). The desirability of the ‘democracy’ label is reflected among citizens as well: in the World Values Surveys, citizens predominantly agree that democracy may have its problems but it is better than other political systems (Zagrebina, 2020). In light of this anecdotal and scholarly evidence, we presume citizens would place themselves on the democratic side. Crucially, we argue that the desirability of the ‘democratic’ label should map onto how they perceive the democraticness of their in-party (i.e. chosen party): voters will perceive their in-party as respecting democratic norms and practices.

In contrast, the opposite scenario applies to the ‘undemocratic’ label: politicians often label rivals as ‘undemocratic’ but not themselves (Lührmann, 2021). For example, after the FBI raided Trump’s residence to search for classified documents, he wrote on social media, ‘This Break In was a sneak attack on democracy (our Republic!)’ (Roche, 2022), which hints that democracy is at stake under Biden’s presidency. Similarly, after Bolsonaro’s loss in the 2022 presidential election, he condemned the left-wing bloc as ‘undemocratic’ for impeding his re-election through electoral fraud (Schedler, 2023). Such labelling of the out-group as ‘undemocratic’ is also found after the 2019 Bolivia election. In the post-election crisis that ended the presidency of Evo Morales – a far-left authoritarian according to scholars (Velasco Guachalla et al., 2021; Weyland, 2013) – he accused the opposition of launching an ‘undemocratic’ coup (Lehoucq, 2020). Given the similarity (and frequency) of these labelling processes, one can conceive that voters are likely to perceive their out-party as being undemocratic. This motivated response to the out-party can also link with the recent literature that finds citizens perceive their country’s quality of democracy as being worse when the out-party is in power (Carey et al., 2019; Flavin and Shufeldt, 2024). Taken together, we expect partisan bias in how citizens evaluate the democratic quality of a party: citizens will perceive their in-party as more likely to respect democratic norms than the out-party(-ies).

## How Do Citizens Update in Light of Undemocratic Behaviour?

Yet, do citizens update a party’s perceived democraticness after exposure to its undemocratic behaviour (according to scholarly standards)? Are perceptions stable or vulnerable to change? We argue that change in the perceived democraticness of a party depends upon citizens’ party preferences, an expectation grounded in the extensive literature on motivated reasoning (Anduiza et al., 2013; Jerit and Barabas, 2012; Lebo and Cassino, 2007; Nyhan and Reifler, 2010; Redlawsk, 2002; Taber and Lodge, 2006). According to this literature, a citizen’s information-updating process is asymmetrical: citizens fail to correct when they are exposed to information unfavourable to their in-party (undemocratic behaviour in our case), while they do update their perception when the unfavourable information concerns an out-party.

To elaborate, we expect that the perceived democraticness of the in-party should remain stable when citizens are exposed to the undemocratic behaviour of the in-party. The rationale behind this is twofold. For one, in-party voters may engage in directional reasoning by discounting information that contradicts their standing beliefs (Jerit and Barabas, 2012; Lebo and Cassino, 2007; Nyhan and Reifler, 2010). For another, in-party voters’ reasoning may be accuracy-motivated and suspect the credibility of information (Druckman and McGrath, 2019). Suppose a Republican governor uses emergency power to repress a peaceful left-wing movement using excessive police force. This act would certainly violate the democratic principle of freedom of expression according to scholarly standards. However, Republican voters may evaluate the information with a directional bias by discarding this new information because it contradicts their prior perceptions (i.e. in-party respects democratic norms and practices). Also, Republican partisans may consider this new information (i.e. the undemocratic behaviour of the in-party) as not credible, for the Republican elites are likely to frame the emergency act as ‘democratic’ and justify this act as representing the national interest. So, even when voters are primed to consider the undemocratic behaviour of their in-party, they will not significantly adjust their in-party’s perception.

In contrast, we expect that the perceived democraticness of the out-party would change in the undemocratic direction when voters are exposed to messages about the out-party’s undemocratic behaviour. As suggested by Druckman and McGrath (2019), citizens are motivated to process new information in a biased manner that strengthens their negative evaluations of an out-party. Moreover, accuracy-motivated citizens may consider the new information as more credible if the information is unfavourable to the out-party. To see how this idea of motivated reasoning maps onto perceived democraticness, we can use the US January 6th Capitol insurrection as an example. According to scholarly standards, this event would certainly be classified as undemocratic for it undermined a free and fair elected government. In such a scenario, we would expect Democrat voters to update the perceived democraticness of the Republican Party for the above two reasons. On the one hand, Democrats would incorporate this new information (i.e. the undemocratic behaviour of the out-party) because it reinforces their prior perceptions. On the other hand, Democrats may also find information unfavourable to the out-party more credible. Taken together, we hypothesize that voters will perceive an out-party as more undemocratic after they are primed with information about the undemocratic behaviour of the out-party.

## Case of Canada

To test the above expectations on motivated responding and asymmetrical information-updating, we study the perceived democraticness of different parties from the Canadian voter’s perspective. There are several reasons why we chose the case of Canada. First, we have a benchmark from experts’ evaluation regarding the democratic quality of different parties. According to experts’ opinions in the 2019 GPS, all Canadian mainstream parties respect liberal democratic principles, norms and practices. On a 0–10 scale, the democratic quality of the main national parties is the following in descending order: New Democratic Party (8.57), Liberal Party (8.54), Green Party (8.40) and Conservative Party (6.57).^5^ If the perceived democraticness of a party demonstrates partisan bias, then we should find disparities between citizens’ perceptions and experts’ evaluations.

Second, from scholars’ perspectives, Canada has been widely regarded as an exemplar of liberal democracy. According to the V-Dem dataset, Canada’s Liberal Democracy Index (LDI) has remained above 0.73 (along a 0–1 scale) since the 1970s.^6^ Public opinion ratings are similar: Leduc and Pammett (2014) report an average ‘democraticness’ score of 7.4 on a 0–10 scale in a 2012 survey.^7^ However, given the events during the pandemic, Canadian scholars have raised concerns about the country’s democratic quality. For one, there is the notion of executive aggrandizement due to the Liberal government’s emergency measures (Gidengil et al., 2022). For another, there is the rise of a populist far-right movement whose supporters are against vaccinations and health restrictions (Chan and Stephenson, 2024). Importantly, even as this far-right movement denied the legitimacy of the elected government during the trucker convoy, some Conservative politicians were observed showing their support for the far-right protesters. The autocratizing behaviour of both mainstream parties provides us with a valuable opportunity to test if citizens’ perceptions update in light of information about these undemocratic behaviours (according to scholarly standards). Our pre-registered experiment leverages this rare opportunity.

Third, we are not aware of any research that studies the perceived democraticness of a party *in a multi-party system*. The closest we can think of is the work by Pasek et al. (2022), whose correlational analysis illustrates that US citizens perceive that their in-party Congressperson respects democratic norms and practices more than the out-party Congressperson. Yet, this study is about a two-party system in which the in-party and out-party are obvious. When it comes to multi-party systems, people may develop their perceived democraticness in more nuanced ways. As scholars studying affective polarization in multi-party systems suggest, what constitutes an in-party or an out-party depends upon ideology (Horne et al., 2023; Kekkonen and Ylä-Anttila, 2021). For instance, voters of a left-wing party may consider other left-wing parties as part of their in-group, whether due to legislative cooperation (such as in coalitions) or because of ideological similarity. This ideological bloc element may induce a halo effect: voters may see a party from the same ideological bloc favourably in terms of its perceived democraticness.

## Data and Research Design

Our data come from a nationally representative sample (n = 1129) run as part of the Democracy Checkup survey in Canada by the Consortium on Electoral Democracy (C-Dem), which was fielded from 5 May to 20 May 2022 (Harell et al., 2023). To ask respondents about the perceived democraticness of different parties, we adapted a question from the 2019 GPS expert survey. The question reads: ‘Some parties are considered as respecting democratic norms and practices, while others are not. On a scale from 0 to 10, where 0 means “democratic” and 10 means “undemocratic”, where would you place each federal party on the following scale?’. Our analysis reverses the scale, such that smaller values mean undemocratic and larger values mean democratic. Respondents were asked to position the main federal parties at the time: the Liberal Party (LPC), the Conservative Party (CPC), the New Democratic Party (NDP), the Green Party (Green) and the People’s Party of Canada (PPC).^8^ We operationalize respondents’ party preference by their chosen party in the 2021 general election (see Table A.1 for survey wordings and Table A.2.1-3 for summary statistics).

It is beyond the scope of our research to analyse citizens’ substantive understanding of this democratic norms and practices scale (for citizens’ understanding of democracy, see Davis et al., 2021; Osterberg-Kaufmann et al., 2020). Yet, we suggest that respondents used the scale in a colloquial way, which reflects how politicians label in-parties and out-parties. Two aspects of the data support this claim. First, there is very little missingness in the data – the highest percentage is below 3% (Table A.3) – which suggests that respondents are able to evaluate the parties even when some are much smaller (and therefore less well-known). Second, as we shall see, the perceived democraticness of different parties follows an ideological pattern, which is in line with our expectations about motivated responding.

Our analysis is separated into two parts. The first part is descriptive, and the second part is experimental. The first part analyses the control group (n = 374) to see if there is evidence of partisan bias in how citizens perceive the democraticness of different parties. If partisan bias is present, all voters should perceive their chosen party (in-party) as more democratic than the out-parties. Also, considering the dynamics of the Canadian multi-party system, we explore whether this motivated response extends within ideological blocs. Accordingly, within the left-wing bloc, LPC voters may perceive the NDP as similarly democratic to the LPC, and vice versa. We identify this ideological bloc because the two left-wing parties hold similar ideological stances (see evidence in Cochrane, 2015; Merkley, 2022) and they formed a confidence and supply agreement in March 2022. Regarding the right-wing bloc, we likewise explore whether CPC voters perceive the PPC as similarly democratic to the CPC, since the PPC leader previously ran for the CPC leadership before leaving the CPC. To test whether the difference between the in-party’s perceived democraticness and that of the out-party is statistically significant, we use one-tailed t-tests as we have strong expectations of motivated responding: the in-party should be perceived as more democratic than an out-party. Also, because the comparisons involve in-parties with various out-parties, we use Holm’s method to deal with the issue of multiple comparisons. In brief, the first part of our analysis is descriptive, and we consider this task worthwhile because no prior works have probed citizens’ perceptions of the democraticness of in-parties and out-parties within a multi-party system.

The second part is a pre-registered experiment that tests whether political messages can change the perceived democraticness of in-parties and out-parties.^9^ Our experiment leverages the 2022 trucker convoy, in which both major mainstream parties (LPC and CPC) engaged in behaviours that would be judged as undemocratic by scholars. We embedded two separate treatments in the experiment, each of which was a real headline from the Canadian media during the 2022 trucker convoy. The first treatment is about the Emergencies Act invoked by the LPC in response to the convoy (see Figure B.1, n _treatment 1_ = 371). Under the Emergencies Act, participation in certain types of public assemblies was prohibited, which, according to the V-Dem indicator ‘State of emergency (v2casoe)’, is undemocratic because the Act could enable state institutions to restrict the behaviour of civil society. The second treatment is about the CPC’s support of the convoy (see Figure B.2, n _treatment 2_ = 384). During the convoy, some far-right extremist groups promoted violence and advocated overthrowing the federal government. According to V-Dem criteria, the CPC’s support for this movement is undemocratic because the convoy was a ‘Mobilization for autocracy (v2caautmob)’, as these far-right actors aimed to undermine a government elected by a free and fair election.

Our pre-registered hypothesis expected an asymmetric information-updating process in line with motivated reasoning. When voters are primed to consider the undemocratic behaviour of their in-party, their perception should remain stable. This means the perceived democraticness of the LPC (or that of the CPC) would remain unchanged when LPC voters (or CPC voters) are exposed to information about the undemocratic behaviour of their in-party. To test for the precision of null results, we conduct equivalence tests suggested by Lakens (2017). In contrast, when voters are primed to consider the undemocratic behaviour of an out-party, they should incorporate this information and perceive the out-party as more undemocratic. Specifically, LPC voters should perceive the CPC as more undemocratic when exposed to the CPC undemocratic prime, and vice versa. The OLS regression includes socio-demographic variables as controls (age, gender, education, province and income).^10^ We exclude respondents who fail the factual manipulation check (n = 46, see Figure B.4 for the manipulation check and Table B.1 for its statistics). According to the pre-registered plan, our main analysis focuses on the treatment effects among LPC voters and CPC voters. The reasons are twofold. First, theoretically, these two parties are the only parties that can viably become the governing party. Thus, considering the size and dominance of these two major parties, it is crucial to understand the effect of out-party undemocratic primes among their voters. Second, empirically, we expected an insufficient sample size for other party voters and abstainers in the data collection. Nevertheless, our exploratory analysis includes testing the treatment effects among NDP voters and abstainers, as their sample size may still be enough to identify an effect.

## Results

### Descriptive: Perceived Democraticness of a Party (Control Group)

**Figure 1.**
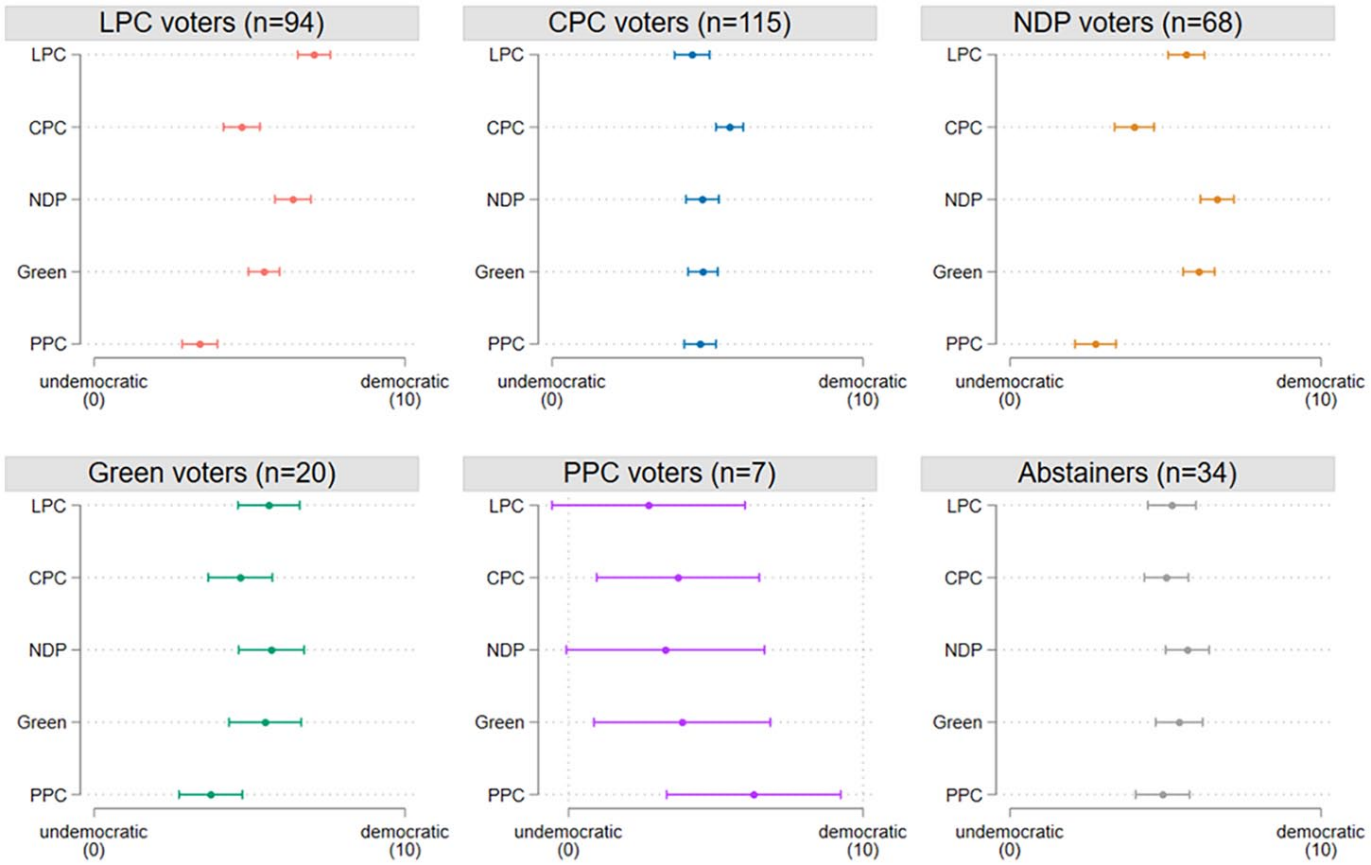
Perceived Democraticness of Different Parties by Voters of Each Party and Abstainers. The estimates show the marginal distribution of placements by vote choice. The dependent variable has a scale of 0–10, where 0 means ‘undemocratic’ and 10 means ‘democratic’. Voters of a party are operationalized by the party that respondents voted for in the 2021 general election; abstainers are those who did not cast votes. Spikes show 95% confidence intervals.

Figure 1 illustrates the perceived democraticness of different parties. The first five panels show the placements by voters of each party and the last panel is the placements by abstainers. To begin, the findings largely confirm our expectation of motivated responding: voters of all three mainstream parties – LPC, CPC and NDP – perceive their in-party as more likely to respect democratic norms and practices than the out-parties (see Table C.1). For voters of more minor parties (Green and PPC), we are less confident about the perceived democraticness of their in-party and out-parties due to the small sample. Nevertheless, for voters of all three mainstream parties, the partisan bias is clear: voters perceive their chosen party as most democratic. This partisan bias is hard to reconcile with how scholars judge the democratic quality of a party.

Next, we elaborate on the ideological pattern of such perceived democraticness. We find a clear pattern in how voters of left-wing and right-wing parties perceive the democraticness of an out-party from the opposite ideological bloc. Specifically, LPC voters perceive the PPC as the party that least respects democratic norms and practices (∆ = 3.71, p = 0.000), followed by the CPC (∆ = 2.33, p = 0.000). The same phenomenon applies to NDP voters: their perceived democraticness of the PPC is smallest (∆ = 3.99, p = 0.000) and then the CPC is the next most undemocratic party (∆ = 2.68, p = 0.000). On the other hand, CPC voters perceive the LPC as the least democratic (∆ = 1.21, p = 0.002). In short, voters of left-wing and right-wing parties both perceive an out-party from the opposite ideological bloc as least democratic.

Last, we analyse whether voters are favourable to other parties in their ideological bloc. Within the left-wing bloc, LPC voters may perceive the NDP as similarly democratic to the LPC, and vice versa, because of their ideological similarity and parliamentary cooperation (Cochrane, 2015; Merkley, 2022). The analysis suggests this is not the case. Although LPC voters perceive the NDP as the second most democratic party, the difference between the perceived democraticness of these two left-wing parties is still statistically significant (∆ = 0.68, p = 0.002). A similar differentiation is also seen among NDP voters in how they perceive their in-party and the LPC (∆ = 1.21, p = 0.001). Regarding the right-wing bloc, we likewise find that CPC voters do not perceive the PPC as similarly democratic to the CPC (∆ = 0.96, p = 0.001). This finding echoes a contrast effect in how voters of a mainstream right party perceive a far-right party (Chan, 2022). Overall, this means voters would still perceive their in-party as more democratic than the other party that belongs to the same ideological bloc.

Taken together, the above analysis demonstrates that the perceived democraticness of in-parties and out-parties involves motivated responding. There are three takeaway points from this descriptive exercise. First, voters of the three mainstream parties perceive their in-party as most democratic, which speaks to the presence of an in-party bias. Second, negative out-party bias is strongest for ideologically opposite parties: voters of left-wing and right-wing parties both perceive an out-party from the opposite ideological bloc as least democratic. Third, even if an out-party belongs to the same ideological bloc, voters still perceive that out-party as less democratic than their in-party. These findings on perceived democraticness hardly match how scholars judge the democratic quality of a party, which has a single absolute ordering pattern.

### Experiment: Causal Effects of the Undemocratic Prime

**Figure 2.**
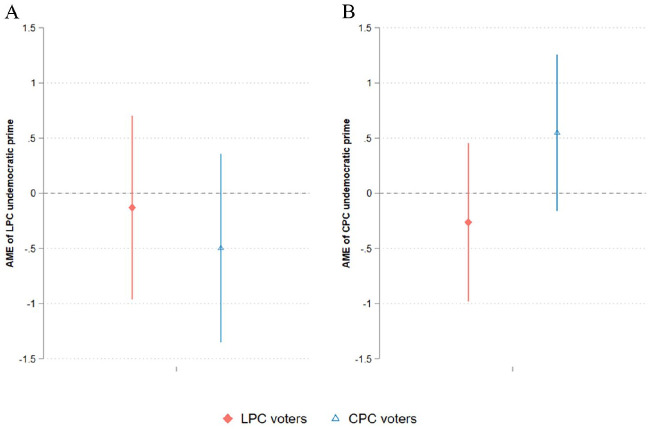
Impact of Exposure to a Party’s Undemocratic Prime. (A) Placement of LPC by LPC and CPC voters. (B) Placement of CPC by LPC and CPC voters. The triangles and diamonds indicate the average marginal effects of exposure to an undemocratic prime versus being in the control group. The dependent variable has a scale ranging from 0 to 10, where 0 means ‘undemocratic’ and 10 means ‘democratic’. Spikes show 95% confidence intervals.

To test whether political messages can change perceptions of democraticness, we turn to Figure 2 which illustrates the findings of our pre-registered experiment (for regression results, see Table C.2). Panel A concerns the placement of the LPC, and the treatment is an LPC undemocratic prime. Panel B concerns the placement of the CPC, and the treatment is a CPC undemocratic prime. To reiterate, invoking the Emergency Act and supporting a pro-autocracy mobilization are undemocratic when judged by scholarly standards. Furthermore, both behaviours received considerable media attention during the pandemic. Our pre-registered hypothesis leverages this episode of democratic backsliding to test for the asymmetrical information-updating process. That is, when voters are exposed to the undemocratic behaviour of their in-party, they should not update their perceived democraticness. In contrast, they should update the undemocratic behaviour of the out-party and perceive it as more undemocratic.

We first discuss the impact of an undemocratic prime on in-party evaluations. As hypothesized, both LPC voters and CPC voters do not significantly change their perceptions when they are exposed to an in-party undemocratic prime. Among CPC voters, we even see a slight indication that the treatment causes them to see their in-party as more democratic instead of less, although the effect is not statistically significant (p = 0.129). To check for the precision of null results, we follow Lakens (2017) and conduct equivalence tests for p < 0.05, assuming unequal variances of the control group and the treatment group. After running these tests, we find that the prime would shift the perceptions of in-party voters towards the undemocratic direction by no more than 0.8 units among LPC voters and 0.12 units among CPC voters (on an 11-point scale). That means, even after providing the undemocratic prime to in-party voters, they do not substantively update the perceived democraticness of their in-party.

Next, we ascertain whether exposure to an out-party undemocratic prime leads to re-evaluations in a way consistent with motivated reasoning. According to our pre-registered hypothesis, the prime should cause CPC voters to perceive the LPC as more undemocratic in panel A, while it should cause LPC voters to perceive the CPC as more undemocratic in panel B. Although both treatment effects are in the expected direction, the effects fail to reach conventional levels of statistical significance (p = 0.252 for LPC undemocratic prime; p = 0.473 for CPC undemocratic prime). In our exploratory analysis of NDP voters and abstainers, we likewise do not find any effects when they are primed about the undemocratic behaviour of the LPC and CPC (Figure C.1 and Table C.3).

However, we suspect these null effects may hide heterogeneity among subgroups (Kane, 2025). This is because the convoy was a wedge issue that divided the CPC, just like whether the US 2020 election was ‘stolen’ is a polarizing issue among Republicans (Arceneaux and Truex, 2023: 13). To test this heterogeneity, we use a pre-treatment moderator variable that asks about respondents’ attitudes towards the trucker convoy. The question asked respondents how much they agreed that ‘the protesters were protecting Canadians’ rights and freedoms’. To be transparent, the exploratory analysis is not pre-registered and this moderator is the first variable that we explored. Yet, we have theoretically strong reasons to conjecture that updating the perceived democraticness of the out-party may be conditioned by voters’ attitudes towards the event: research on motivated reasoning suggests that prior attitudes on an issue can strengthen/offset how partisans update a piece of information (Mullinix, 2016). Applying this idea to our case, incorporating the undemocratic behaviour of the out-party may not depend simply on party preferences, but also on voters’ prior attitudes towards the trucker convoy.

**Figure 3.**
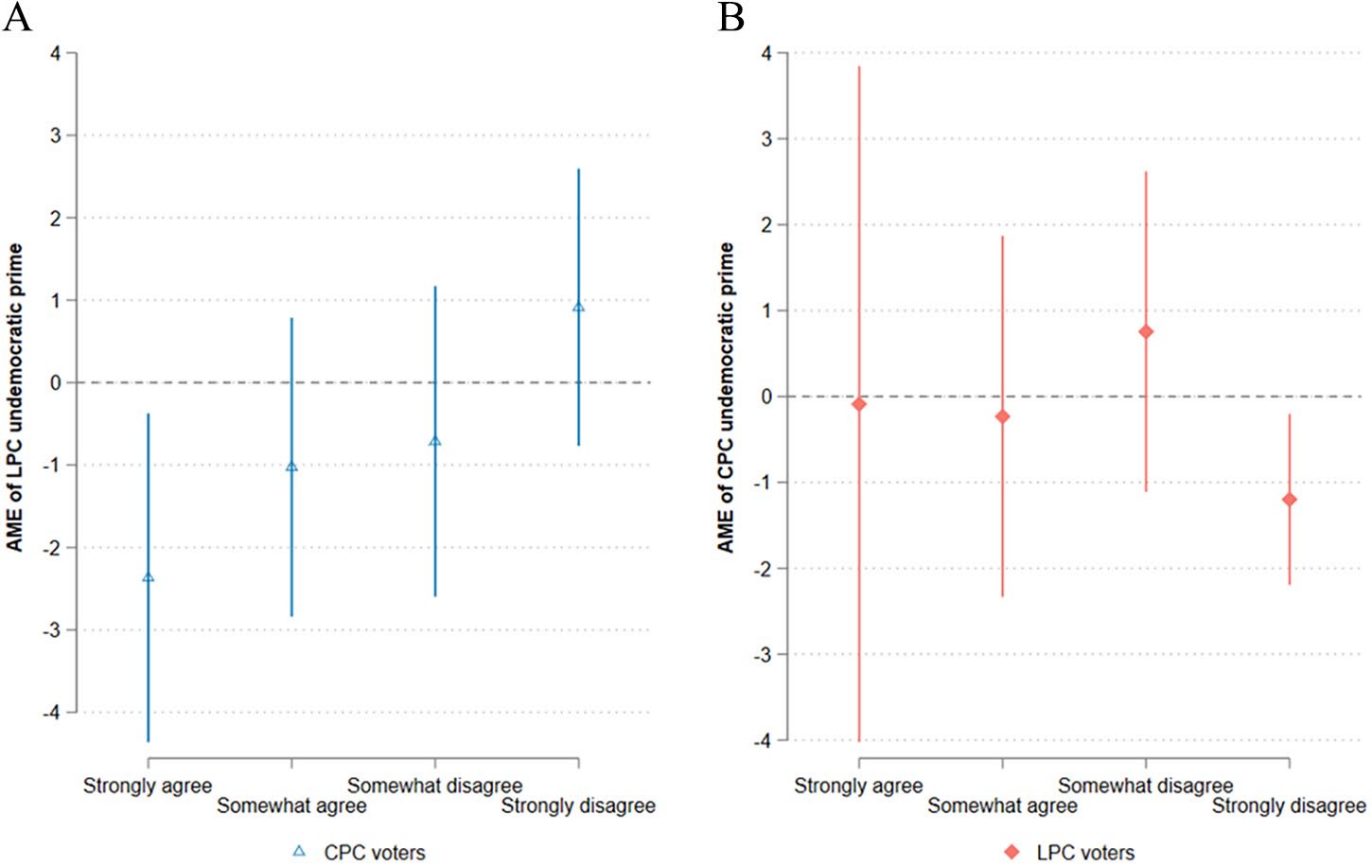
Moderating Effect of Attitude Towards the Convoy. (A) Placement of LPC by CPC voters. (B) Placement of CPC by LPC voters. The triangles and diamonds indicate the average marginal effects of exposure to an undemocratic prime versus being in the control group. The dependent variable has a scale ranging from 0 to 10, where 0 means ‘undemocratic’ and 10 means ‘democratic’. In panels A and B, the moderator variable has a four-point ordinal scale, which asks respondents how much they agree ‘the protesters were protecting Canadians’ rights and freedoms’. Spikes show 95% confidence intervals.

The exploratory analysis in Figure 3 confirms the importance of prior attitudes towards the event and the hidden heterogeneity (for regression results, see Table C.4). Panel A shows that the LPC undemocratic prime causes CPC voters to perceive the LPC as more undemocratic when they strongly agree that the convoy protects Canadians’ rights and freedoms. The magnitude of the treatment effect is 2.37 units along an 11-point scale (SD = 0.749, p = 0.020), which is substantively large. Similarly, in panel B, LPC voters perceive the CPC as more undemocratic by 1.20 units when they strongly disagree that the convoy protects rights and freedoms (SD = 0.431, p = 0.019).^11^ As a sensitivity analysis, we recode the moderator into a binary variable (‘Strongly agree’ and ‘Somewhat agree’ = 0; ‘Somewhat disagree’ and ‘Strongly disagree’ = 1). We still identify a moderating effect among CPC voters, while the moderating effect among LPC voters ceases to become significant (Figure C.2 and Table C.5). In other words, the moderating effect is only manifest among respondents who hold strong opinions about the convoy protecting rights and freedoms.^12^

In short, this experimental evidence uncovers an asymmetrical information-updating process regarding how voters update a party’s perceived democraticness. While voters fail to correct their perceptions of the in-party’s democraticness when exposed to its undemocratic behaviour, they do update when the undemocratic behaviour concerns an out-party in certain situations. Specifically, an undemocratic prime about an out-party can reduce the out-party’s perceived democraticness when voters hold strong opinions about the democratic quality of an event. More broadly, this indicates that conditional on voters’ prior attitudes, political messages can change citizens’ perceptions of whether an out-party is democratic or not.

## Conclusion

Scholars often make claims about the democratic quality of various parties (Düpont et al., 2022; Medzihorsky and Lindberg, 2024). Yet, there is little focus on the perceived democraticness of parties from a citizen’s perspective. This void is curious because politicians often label themselves as ‘democratic’ and out-party candidates as ‘undemocratic’. Building upon the motivated reasoning literature, our article argues that this labelling process should map onto citizens’ perceptions even in a well-regarded liberal democracy. First, our descriptive findings demonstrate motivated responding: people perceive their in-party as more committed to democratic norms than the out-party(-ies). Second, leveraging the 2022 trucker convoy, we test the vulnerability of such perceptions by priming a party’s undemocratic behaviour (according to scholarly standards). The experimental study uncovers an asymmetrical information-updating process. For one, voters do not update their perceptions of their in-party when primed with the in-party’s undemocratic behaviour. For another, voters who receive a similar prime about the out-party perceive that party as more undemocratic *if* they hold strong opinions about the convoy. Still, readers should note that we do not find support for our pre-registered hypothesis concerning the effect of an out-party prime. Rather, the effect of the out-party prime we observe comes from an exploratory analysis based on the motivated reasoning literature.

Overall, our research should enrich the partisan bias literature and studies on democratic backsliding. Scholars identify that partisan bias can weaken democratic accountability and this bias exists in various domains, such as economic assessments (Anderson et al., 2004; Ansolabehere et al., 2013) and corruption scandals (Anduiza et al., 2013; Pereira Filho et al., 2024). Recently, research on democratic backsliding has adopted this perspective to explain why partisans hold divergent assessments on the quality of their domestic democracy (Carey et al., 2019; Flavin and Shufeldt, 2024), the legitimacy of an election (Arceneaux and Truex, 2023) and politicians’ undemocratic behaviour (Krishnarajan, 2022). Our research joins this endeavour but departs from these works by asking about citizens’ perceived democraticness of different parties and testing the vulnerability of these perceptions. We consider this domain – the perceived democraticness of a party – important because, in daily political news, parties emphasize how their behaviour aligns with ‘democracy’ and criticize the out-party(-ies) as ‘undemocratic’. Although the asymmetrical information-updating process that we uncover aligns with the partisan bias literature, the implications are disconcerting. In the face of undemocratic behaviour (by scholarly standards), an ideal situation would be that citizens are neutral arbiters and update their perception of the in-party as much as that of the out-party. Our findings suggest this ideal scenario hardly exists even in a well-regarded liberal democracy. This asymmetrical information-updating process may explain why interventions fail to reduce partisans’ support for violence and bolster their support for democratic norms (Dias et al., 2024; Wuttke et al., 2024).

We see four major extensions that future studies can undertake. The first concerns the measures of our outcome. Our research merely focuses on citizens’ perceptions of different parties. Yet, citizens can similarly position *politicians* on this scale. For instance, do MAGA Republicans position Trump and other Republican politicians (e.g. Liz Cheney, Mike Pence) at a similar point to the party itself? The overlap/deviation between party and candidates on this scale is an exciting question to consider because the difference can denote dissonance within a party. We can also foresee that this difference should be relevant for research on partisan support for violence (Mernyk et al., 2022). More broadly, the scale that we adapt from the GPS is tapping into citizens’ perceived democraticness of parties, not their degrees of authoritarianism. Though scholars always put ‘democracy’ and ‘authoritarianism’ on a single scale, these two notions may trigger different associations (for a similar discussion on left-right, see Bauer et al., 2017). Therefore, subsequent works can change the wording of the one pole from ‘undemocratic’ to ‘authoritarian’ to see if respondents answer differently and investigate what they are thinking concretely. This could be done by providing respondents with an open-ended response after asking about the perceived democraticness of a party.

Second, our article delineates two lines of motivated reasoning that explain why the information-updating process of the in-party’s and out-party’s undemocratic behaviour is asymmetrical. One is directional motivation, which suggests a voter’s goal when incorporating information is to arrive at a predetermined conclusion. Accordingly, voters disregard the in-party’s undemocratic behaviour and only incorporate the out-party’s undemocratic behaviour, since the new information should be consistent with their prior belief (i.e. in-party is democratic while the out-party is not). Another is accuracy motivation, which concerns the credibility of the information. In this case, citizens do learn from the new information, and they simply suspect the credibility of the in-party’s undemocratic behaviour or magnify the out-party’s undemocratic behaviour. Both motivated reasoning mechanisms can give rise to the same observations that support the asymmetrical information-updating process. Our experiment does not disentangle the two motivations, and future research may try to manipulate directional motivation and accuracy motivation separately.

The third extension concerns the treatment of the experiment. Our experiment provided respondents with a prime about a major mainstream party’s behaviour that scholars would judge as undemocratic. Yet, as underlined in this article, politicians are not fully passive and can take part in framing such behaviour as well. For instance, in a study of the US January 6th Capitol insurrection, scholars find that Democratic congresspersons were likely to describe the event as an assault on democracy, while Republican congresspersons were more prone to portray it as a ‘protest grown out of hand’ (Craig and Albertson, 2024). Considering that politicians’ framings of what would be judged as undemocratic behaviour (according to scholarly standards) are also divided along partisan lines, future studies can test whether and how such framings affect the perceived democraticness of a party.

The last extension concerns the context of this study. Our case – Canada – is a liberal democratic regime that has a majoritarian political system. In majoritarian democracies, it is common for one of the two major mainstream parties to form the government, and partisan bias is highly expected. As such, it is worthwhile to test whether our descriptive and experimental findings are generalizable to consensus democracies, in which coalition government is the norm. As consensus democracies always require cooperation between parties, we might expect the motivated response to the perceived democraticness of different parties to be attenuated. This expectation has not yet been tested. On the other hand, subsequent studies could also investigate other regime types. If voters in a liberal democracy perceive an out-party as undemocratic, even though the party is considered democratic by scholars, could the opposite scenario apply in non-democratic regimes? That is, could citizens perceive a party as democratic, even though it is judged authoritarian by scholarly standards? If so, this may explain why voters of such parties (e.g. Erdoğan’s AKP in Turkey or Orbán’s Fidesz in Hungary) tolerate and even support these parties, which scholars judge to be authoritarian.

## Supplemental Material

sj-docx-1-psx-10.1177_00323217251324025 – Supplemental material for Perceived Democraticness of Parties From Citizens’ Perspectives: Evidence From Canada Supplemental material, sj-docx-1-psx-10.1177_00323217251324025 for Perceived Democraticness of Parties From Citizens’ Perspectives: Evidence From Canada by Ka Ming Chan and Laura B Stephenson in Political Studies

## Notes

1. The original GPS scale ranges from 0 to 10, where 0 means ‘Strongly respects liberal democratic principles, norms and practices’ and 10 means ‘Strongly undermines liberal democratic principles, norms and practice’. To avoid confusion, *our paper reverses the scale so that smaller values mean undermining democratic norms and practices, while larger values mean respecting them*. After the reversal, all mainstream parties in Germany score above 7.5: Social Democratic Party (8.93); Christian Democratic Union (8.57); Free Democratic Party (9); and Greens (9.5). In contrast, the score of AfD is 0.75. We thank a reviewer for this idea.

2. In the case description section, we elaborate on the experts’ ratings of different parties.

3. The criteria concerns (a) ‘State of emergency (V-Dem indicator: v2casoe)’ that enables state institutions to restrict the behaviour of civil society and (b) ‘Mobilization for autocracy (V-Dem indicator: v2caautmob)’ that undermines a free and fair elected government. More details are discussed in the research design section.

4. Experts rated Likud as 2.91 in the 2019 GPS dataset on a 0–10 scale. Conceivably, after the recent judicial reform (Gidron, 2023), scholars would evaluate this right-wing party as even more undemocratic.

5. Likewise, in the V-Party dataset, all mainstream parties are coded as committed to free and fair elections and reject using violence against political opponents. The People’s Party of Canada (PPC) – a populist far-right party – is not coded in these expert surveys since it is a newer party that only gained real prominence during the pandemic crisis.

6. The LDI denotes to what extent the ideal of liberal democracy is achieved, where 1 is the maximum value. According to this ideal, a regime should have constitutionally protected civil liberties, a strong rule of law, an independent judiciary, and effective constraints on the exercise of executive power (see Coppedge et al., 2016). Canada’s LDI is comparable to most Western European and Scandinavian countries.

7. That survey asked respondents ‘In your opinion, how democratic is Canada?’. The answer is an 11-point scale for which 0 denotes ‘not at all democratic’ and 10 signifies ‘fully democratic’.

8. Respondents were also asked to place the Bloc Québécois (BQ), a regionalist party. We have excluded that party from our analysis here because BQ only runs in the province of Quebec, and hence the majority of the sample would be less familiar with the party.

9. The URL of our pre-registered analysis plan on Open Science Framework (OSF) is: https://osf.io/m5gp7/?view_only=8727e4a43a1e419f9f436238eb3e1b5a.

10. Covariate balance tests show that the control and treatment groups are symmetrical for all socio-demographics (Figures B.3.1 and B.3.2).

11. The LPC voters who strongly disagree that ‘the protesters were protecting Canadians’ rights and freedoms’ constitute 62.5% of the LPC voters in our sample. On the other hand, CPC voters who strongly agree that ‘the protesters were protecting Canadians’ rights and freedoms’ constitute 21.7% of the CPC voters.

12. Our additional analysis also explores if there are any moderating effects among NDP voters and abstainers. We find no moderating effects (Figure C.3 and Table C.6).
