# Supplementary material for: Perceived Democraticness of Parties From Citizens’ Perspectives: Evidence From Canada
Source: Polit Stud (Oxf). 2025 Mar 15;74(1):398–414. doi: 10.1177/00323217251324025 (PMC12875433; doi:10.1177/00323217251324025)
Supplement: sj-docx-1-psx-10.1177_00323217251324025 – Supplemental material for Perceived Democraticness of Parties From Citizens’ Perspectives: Evidence From Canada [file sj-docx-1-psx-10.1177_00323217251324025.docx]

Table of Contents

[Part A. Data description 2](#_Toc179792262)

[Section A.1 Sampling procedures 2](#_Toc179792263)

[Table A.1. Question wording & operationalization of variables 3](#_Toc179792264)

[Table A.2.1 Summary statistics of vote choice 6](#_Toc179792265)

[Table A.2.2 Summary statistics of the continuous and ordinal variables 6](#_Toc179792266)

[Table A.2.3 Summary statistics of the region of residence 6](#_Toc179792267)

[Table A.3. Missing rates of perceived democraticness 7](#_Toc179792268)

[Part B. Experimental setup 8](#_Toc179792269)

[Figure B.1. Treatment of LPC undemocratic prime 8](#_Toc179792270)

[Figure B.2. Treatment of CPC undemocratic prime 8](#_Toc179792271)

[Figure B.3.1 Covariate balance tests (Treatment of LPC undemocratic prime vs. Control) 9](#_Toc179792272)

[Figure B.3.2 Covariate balance tests (Treatment of CPC undemocratic prime vs. Control) 10](#_Toc179792273)

[Figure B.4. Manipulation check 11](#_Toc179792274)

[Table B.1. Statistics of the manipulation check 11](#_Toc179792275)

[Part C. Additional tables and figures 12](#_Toc179792276)

[Table C.1. Distribution of perceived democraticness by vote choice and paired comparisons between in-party and out-parties (Control group only) 12](#_Toc179792277)

[Table C.2. Treatment effects of exposure to a party's undemocratic prime (LPC voters and CPC voters) 13](#_Toc179792278)

[Figure C.1. Impact of exposure to a party’s undemocratic prime (NDP voters and Abstainers) 14](#_Toc179792279)

[Table C.3. Treatment effects of exposure to a party's undemocratic prime (NDP voters and Abstainers) 15](#_Toc179792280)

[Table C.4. Moderating effect of attitude towards the convoy (LPC voters and CPC voters) 16](#_Toc179792281)

[Figure C.2. Recoding the moderator to binary (LPC voters and CPC voters) 17](#_Toc179792282)

[Table C.5. Recoding the moderator to binary (LPC voters and CPC voters) 18](#_Toc179792283)

[Figure C.3. Moderating effect of attitude towards the convoy (NDP voters and Abstainers) 19](#_Toc179792284)

[Table C.6. Moderating effect of attitude towards the convoy (NDP voters and Abstainers) 20](#_Toc179792285)

# Part A. Data description

## Section A.1 Sampling procedures

The 2022 Democracy Checkup (2022 DC) was an online survey of Canadians fielded from May 5th to May 20th, 2022. It procured an online sample of 9829 members of the Canadian general population through Leger Opinion Panel. The Democracy Checkup 2022 was collected in two waves, the “General Population” wave (n=7709) which sampled the general population of Canada, and the “Quebec Oversample” wave (n=2120) which provided an additional sample of the population of Quebec. The two waves ran simultaneously. The survey of our module (n=1129) was embedded in the 2022 DC, and all questions were placed after the main survey. The survey instrument was presented on the Qualtrics online platform.

The “General Population” wave had independent quotas for age category, gender, and province, based on the 2016 Canadian Census. The quota for Quebec was divided into English-language and French-language components. The “Quebec Oversample” wave had independent quotas for age category, gender, and language, which are based on the 2016 Canadian Census for Quebec. Respondents need to be 18 years of age or older and Canadian citizens or permanent residents to participate.

### Table A.1. Question wording & operationalization of variables

| **Variable (name in the dataset)** | **Question wording** | **Answer and coding** |
| --- | --- | --- |
| Perceived democraticness  (lpc_demo, cpc_demo, ndp_demo, gr_demo, ppc_demo) | Some parties are considered as respecting democratic norms and practices, while others are not. On a scale from 0 to 10, where 0 means “democratic” and 10 means “undemocratic”, where would you place each federal party on the following scale?   - Liberal Party - Conservative Party - NDP - Bloc Québécois - Green Party - People's Party | Democratic (0)  1, 2, 3, 4, 5, 6, 7, 8, 9,  Undemocratic (10)  * Our analysis reverses the scale |
| Turnout (dc22_turnout_2021) | Did you happen to vote in the last federal election in 2021? | Yes (1)  No (2)  * those who answered “No” is coded as abstainers |
| Vote choice (vote_2021) | Which party did you vote for in the federal election in 2021? | Liberal Party (1)  Conservative Party (2)  NDP (3)  Bloc Québécois (4)  Green Party (5)  Another party (please specify) (6)  People's Party (7) |
| Attitude to convoy (freedom) | Earlier this year, there were protests in Ottawa and elsewhere as part of the trucker convoy. How much do you agree or disagree with the following statements?  The protesters were protecting Canadians’ rights and freedoms. | Strongly agree (1)  Somewhat agree (2)  Somewhat disagree (3)  Strongly disagree (4) |
| Age (age) | To make sure we are talking to a cross-section of Canadians, we need to get a little information about your background. First, how old are you? | Age is calculated by deducting the year of birth from the present year |
| Gender (gender) | Are you...? | A man (1)  A woman (2)  Non-binary (3)  Another gender, please specify: (4) |
| Education (edu) | What is the highest level of education that you have completed? | No schooling (1)  Some elementary school (2)  Completed elementary school (3)  Some secondary/ high school (4)  Completed secondary/ high school (5)  Some technical, community college, CEGEP, College Classique (6)  Completed technical, community college, CEGEP, College Classique (7)  Some university (8)  Bachelor's degree (9)  Master's degree (10)  Professional degree or doctorate (11)  (1)-(8) are coded as 0 “Without higher education degree”; (9)-(11) are coded as 1 “With higher education degree”. |
| Region of residence (province) | Which province or territory are you currently living in? | Alberta (1)  British Columbia (2)  Manitoba (3)  New Brunswick (4)  Newfoundland and Labrador (5)  Northwest Territories (6)  Nova Scotia (7)  Nunavut (8)  Ontario (9)  Prince Edward Island (10)  Quebec (11)  Saskatchewan (12)  Yukon (13) |
| Income (income) | What was your total household income, before taxes, for the year 2021? Be sure to include income from all sources | No income (1)  $1 to $30,000 (2)  $30,001 to $60,000 (3)  $60,001 to $90,000 (4)  $90,001 to $110,000 (5)  $110,001 to $150,000 (6)  $150,001 to $200,000 (7)  More than $200,000 (8) |

### Table A.2.1 Summary statistics of vote choice

| **Variable** | **Obs** | **Per cent** |
| --- | --- | --- |
| LPC voters | 317 | 28.08 |
| CPC voters | 301 | 26.66 |
| NDP voters | 226 | 20.02 |
| Green voters | 44 | 3.75 |
| PPC voters | 31 | 2.75 |
| Abstainers | 138 | 11.87 |
| Missing | 76 | 6.73 |
| Total | 1053 | 100.00 |

### Table A.2.2 Summary statistics of the continuous and ordinal variables

| **Variable** | **Obs** | **Mean** | **Std. Dev.** | **Min** | **Max** |
| --- | --- | --- | --- | --- | --- |
| lpc_demo | 1109 | 5.55 | 2.99 | 0.00 | 10.00 |
| cpc_demo | 1108 | 4.96 | 2.65 | 0.00 | 10.00 |
| ndp_demo | 1107 | 5.75 | 2.84 | 0.00 | 10.00 |
| gr_demo | 1105 | 5.50 | 2.45 | 0.00 | 10.00 |
| ppc_demo | 1103 | 4.16 | 2.95 | 0.00 | 10.00 |
| freedom | 1113 | 2.95 | 1.10 | 1.00 | 4.00 |
| age | 1129 | 48.74 | 16.76 | 18.00 | 87.00 |
| gender | 1129 | 1.56 | 0.51 | 1.00 | 3.00 |
| edu | 1129 | 0.42 | 0.49 | 0.00 | 1.00 |
| income | 1125 | 4.29 | 1.75 | 1.00 | 8.00 |
|  | | | | | |

### Table A.2.3 Summary statistics of the region of residence

| **Variable** | **Obs** | **Per cent** |
| --- | --- | --- |
| Alberta | 150 | 13.29 |
| British Columbia | 152 | 13.46 |
| Manitoba | 34 | 3.01 |
| New Brunswick | 19 | 1.68 |
| Newfoundland and Labrador | 14 | 1.24 |
| Nova Scotia | 26 | 2.30 |
| Nunavut | 1 | 0.09 |
| Ontario | 501 | 44.38 |
| Prince Edward Island | 3 | 0.27 |
| Quebec | 192 | 17.01 |
| Saskatchewan | 37 | 3.28 |
| Total | 1129 | 100.00 |

### Table A.3. Missing rates of perceived democraticness

| **Variable** | **% of missing (Total sample)** | **% of missing (Control group)** | **% of missing (Treatment groups combined)** |
| --- | --- | --- | --- |
| Placement of LPC | 1.77 | 1.87 | 1.72 |
| Placement of CPC | 1.86 | 2.14 | 1.72 |
| Placement of NDP | 1.95 | 2.41 | 1.72 |
| Placement of Green | 2.13 | 2.41 | 1.99 |
| Placement of PPC | 2.30 | 2.94 | 1.99 |
| Number of observations | 1129 | 374 | 755 |

# Part B. Experimental setup

### Figure B.1. Treatment of LPC undemocratic prime


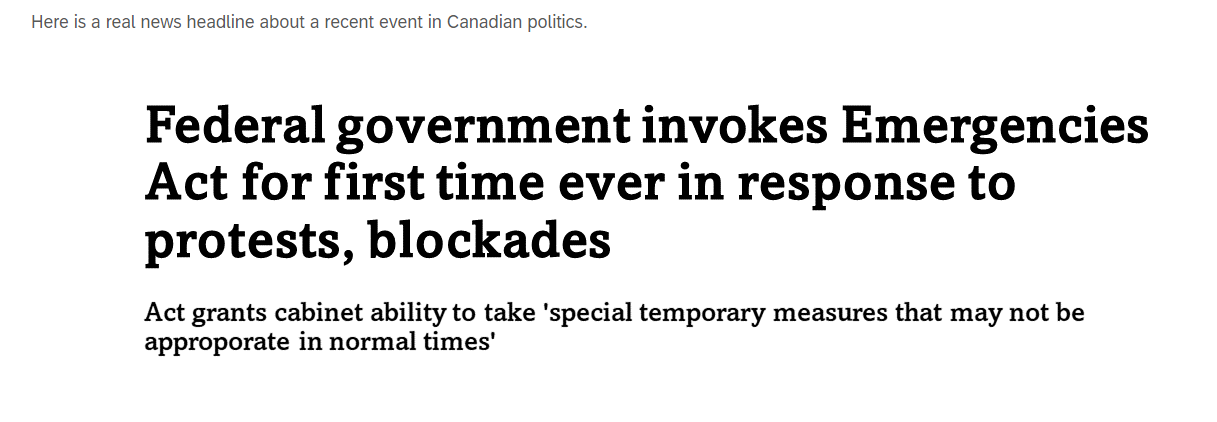


### Figure B.2. Treatment of CPC undemocratic prime


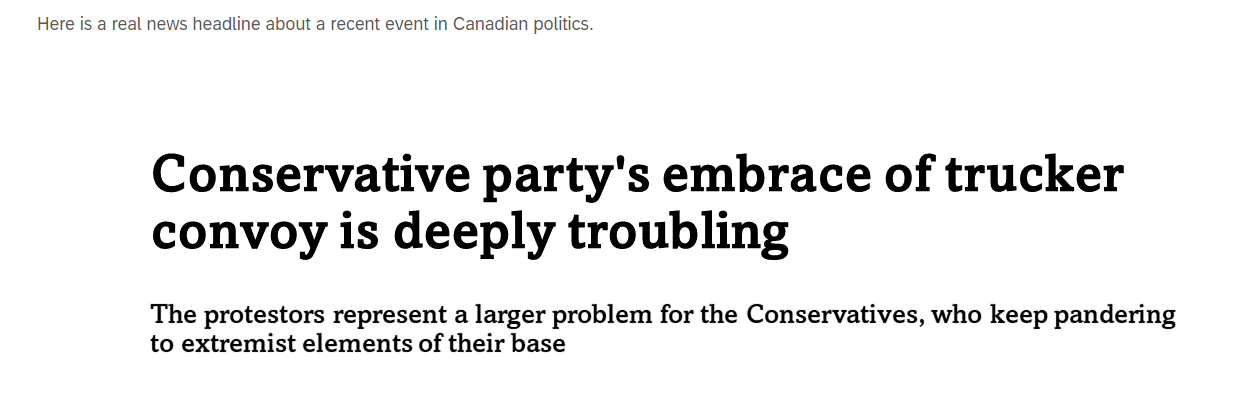


### Figure B.3.1 Covariate balance tests (Treatment of LPC undemocratic prime vs. Control)


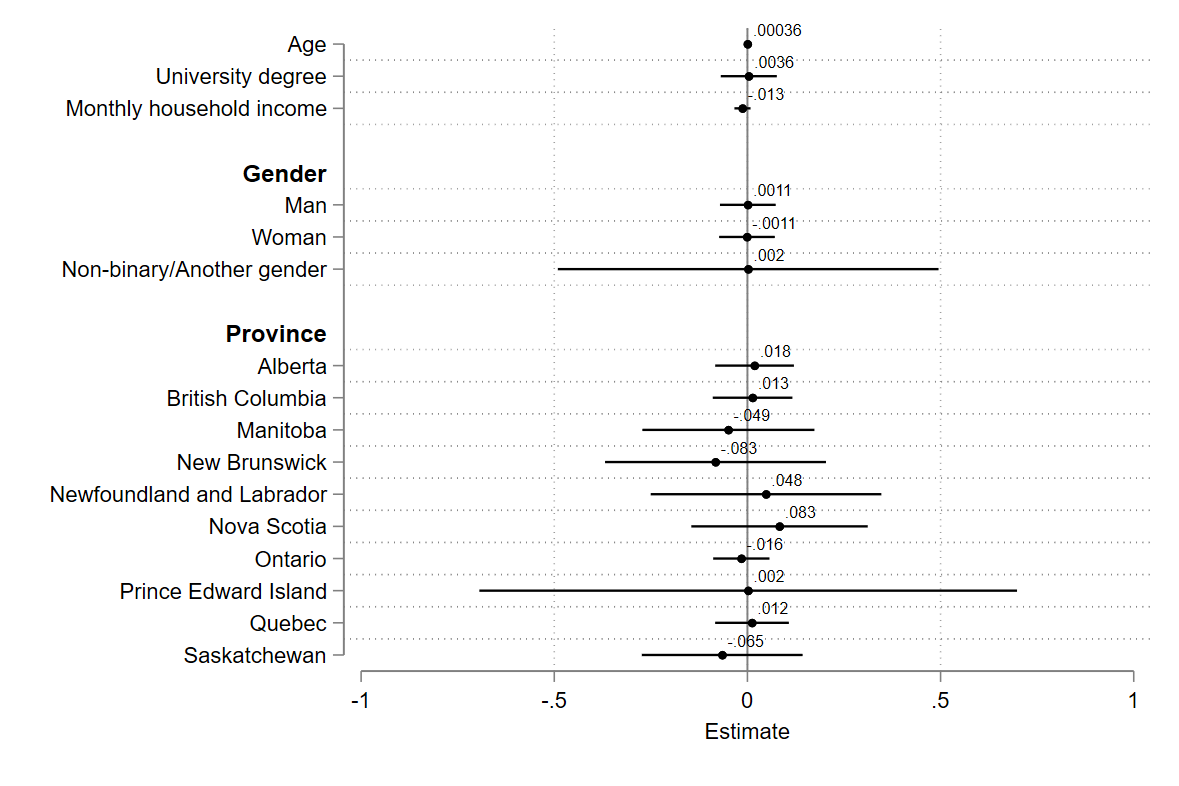


Note: The coefficient plot shows the estimates of OLS models that regress treatment assignment on the observed socio-demographic variables. Horizontal lines represent 95% confidence intervals.

### Figure B.3.2 Covariate balance tests (Treatment of CPC undemocratic prime vs. Control)


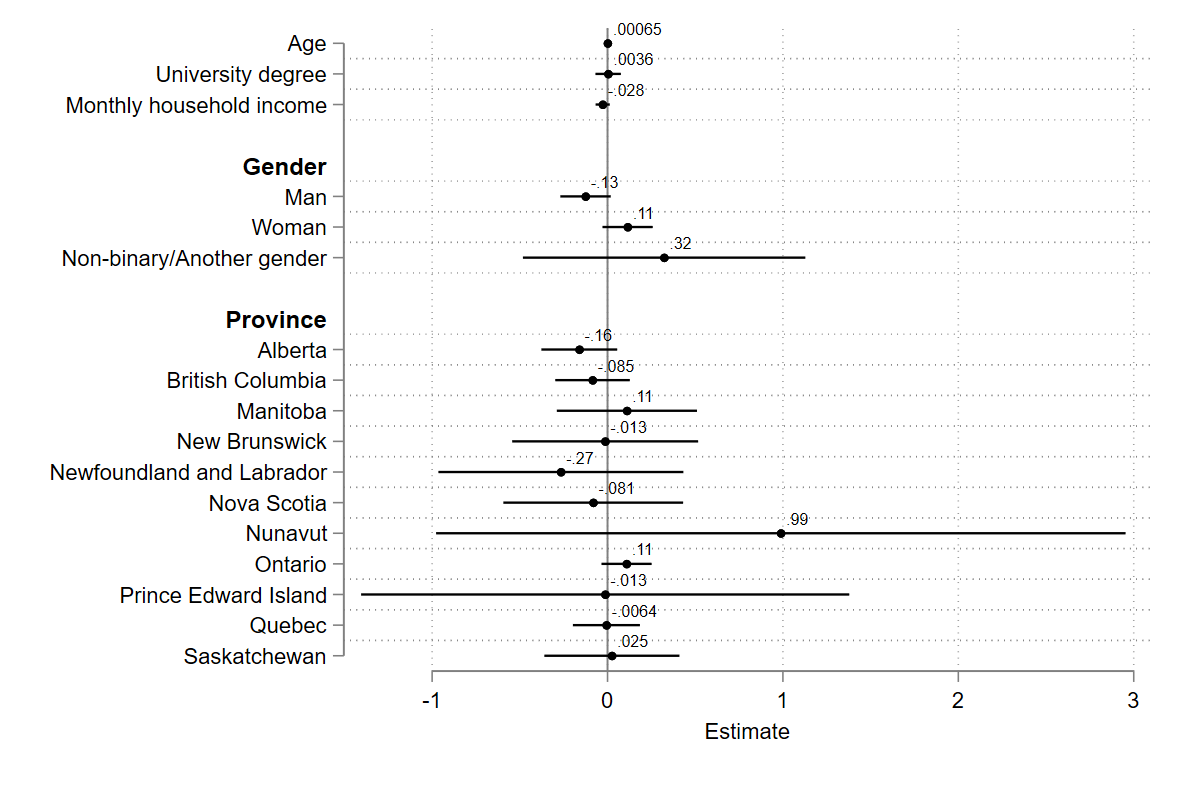


Note: The coefficient plot shows the estimates of OLS models that regress treatment assignment on the observed socio-demographic variables. Horizontal lines represent 95% confidence intervals.

### Figure B.4. Manipulation check

What was the news headline about?

- NDP’s confidence-and-supply agreement with Liberal Party in 2022. (1)
- The convoy protest in early 2022. (2)
- Flooding in British Columbia in late 2021. (3)

### Table B.1. Statistics of the manipulation check

|  | **Percentage of respondents (N)** |
| --- | --- |
| **Passing the manipulation check** | 93.77% (708) |
| **Failing the manipulation check** | 6.10% (46) |
| **No answers** | 0.13% (1) |
| **Total** | 100% (755) |

# Part C. Additional tables and figures

### Table C.1. Distribution of perceived democraticness by vote choice and paired comparisons between in-party and out-parties (Control group only)

|  | **mean** | **Difference with in-party** | **t-stat** | **p-value** |
| --- | --- | --- | --- | --- |
| **LPC voters (n=94)** |  |  |  |  |
| Placement of LPC | 7.07 | n.a. | n.a. | n.a. |
| Placement of CPC | 4.74 | 2.33 | 6.47 | **0.0000** |
| Placement of NDP | 6.39 | 0.68 | 3.00 | **0.0017** |
| Placement of Green | 5.46 | 1.61 | 5.70 | **0.0000** |
| Placement of PPC | 3.40 | 3.71 | 8.79 | **0.0000** |

**CPC voters (n=115)**

| Placement of LPC | 4.50 | 1.21 | 3.01 | **0.0016** |
| --- | --- | --- | --- | --- |
| Placement of CPC | 5.71 | n.a. | n.a. | n.a. |
| Placement of NDP | 4.83 | 0.88 | 2.33 | **0.0107** |
| Placement of Green | 4.85 | 0.86 | 2.44 | **0.0081** |
| Placement of PPC | 4.76 | 0.96 | 3.10 | **0.0012** |

**NDP voters (n=68)**

| Placement of LPC | 5.68 | 0.99 | 3.10 | **0.0014** |
| --- | --- | --- | --- | --- |
| Placement of CPC | 3.99 | 2.68 | 6.30 | **0.0000** |
| Placement of NDP | 6.66 | n.a. | n.a. | n.a. |
| Placement of Green | 6.09 | 0.57 | 2.18 | **0.0163** |
| Placement of PPC | 2.72 | 3.99 | 8.53 | **0.0000** |

**Green voters (n=20)**

| Placement of LPC | 5.65 | -0.15 | -0.44 | 0.6669 |
| --- | --- | --- | --- | --- |
| Placement of CPC | 4.70 | 0.80 | 1.10 | 0.1415 |
| Placement of NDP | 5.70 | -0.20 | -0.39 | 0.6501 |
| Placement of Green | 5.50 | n.a. | n.a. | n.a. |
| Placement of PPC | 3.75 | 1.75 | 2.40 | 0.0134 |

**PPC voters (n=7)**

| Placement of LPC | 2.71 | 3.57 | 1.45 | 0.0982 |
| --- | --- | --- | --- | --- |
| Placement of CPC | 3.71 | 2.57 | 1.36 | 0.1111 |
| Placement of NDP | 3.29 | 3.00 | 1.18 | 0.1415 |
| Placement of Green | 3.86 | 2.43 | 1.12 | 0.1529 |
| Placement of PPC | 6.29 | n.a. | n.a. | n.a. |

**Abstainers (n=34)**

| Placement of LPC | 5.21 | n.a. | n.a. | n.a. |
| --- | --- | --- | --- | --- |
| Placement of CPC | 5.03 | n.a. | n.a. | n.a. |
| Placement of NDP | 5.71 | n.a. | n.a. | n.a. |
| Placement of Green | 5.44 | n.a. | n.a. | n.a. |
| Placement of PPC | 4.91 | n.a. | n.a. | n.a. |

Note: One-sided t-test is used. When the difference between the in-party’s perceived democraticness and that of the out-party is significant at 0.05 level using Holm’s method, p-value is bolded.

### Table C.2. Treatment effects of exposure to a party's undemocratic prime (LPC voters and CPC voters)

|  | (1) Placement of LPC | (2) Placement of CPC |
| --- | --- | --- |
| **LPC undemocratic prime** |  |  |
| LPC voters | -0.13 (0.42) |  |
| CPC voters | -0.50 (0.43) |  |
| **CPC undemocratic prime** |  |  |
| LPC voters |  | -0.26 (0.37) |
| CPC voters |  | 0.55 (0.36) |
| Observations | 389 | 408 |
| Controls | Yes | Yes |

Note: Entries are the average marginal effect of exposure to a party's undemocratic prime. Standard errors are shown in parentheses. The dependent variable has a scale ranging from 0-10, where 0 means 'undemocratic' and 10 means 'democratic'. Controls (age, gender, province, education, education, and income) are omitted from the table.

^+^ *p* < 0.10, ^*^ *p* < 0.05, ^**^ *p* < 0.01, ^***^ *p* < 0.001

### Figure C.1. Impact of exposure to a party’s undemocratic prime (NDP voters and Abstainers)


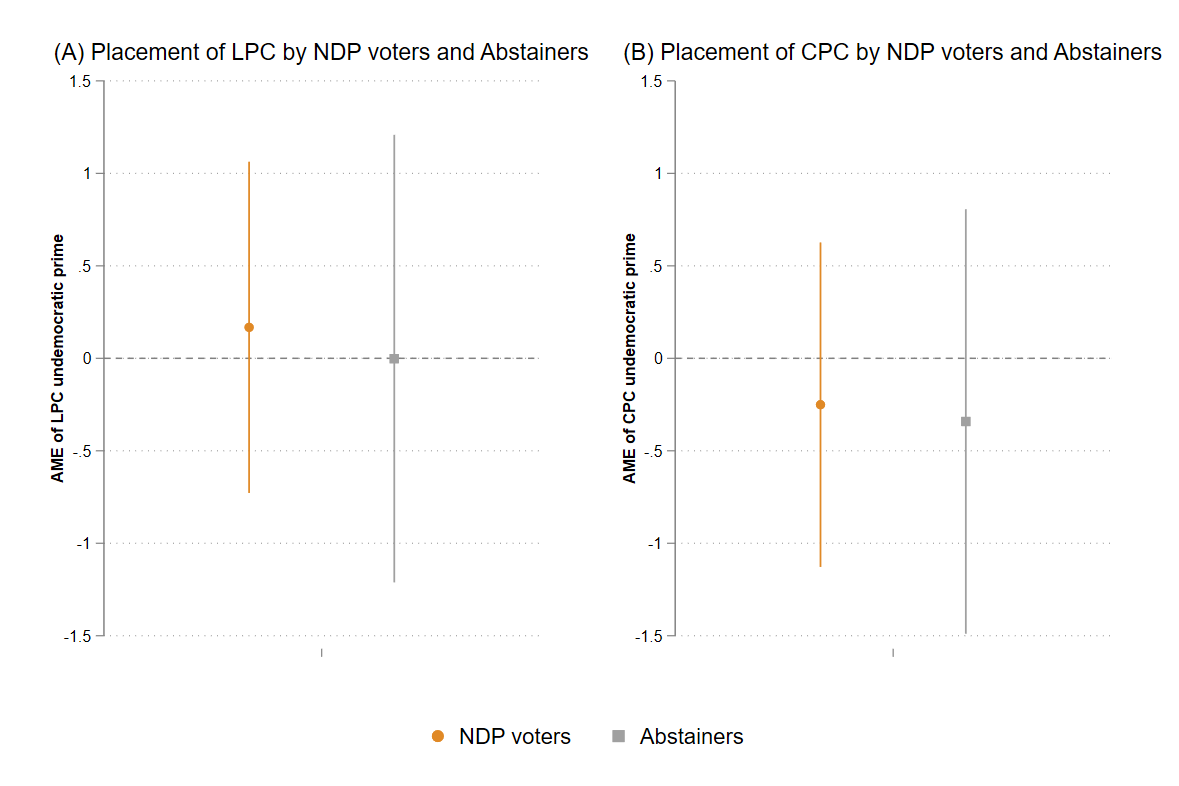


Note: The dot and square indicate the average marginal effect of exposure to an undemocratic prime versus being in the control group. The dependent variable has a scale ranging from 0-10, where 0 means ‘undemocratic’ and 10 means ‘democratic’. Spikes show 95% confidence intervals.

### Table C.3. Treatment effects of exposure to a party's undemocratic prime (NDP voters and Abstainers)

|  | (1) Placement of LPC | (2) Placement of CPC |
| --- | --- | --- |
| **LPC undemocratic prime** |  |  |
| NDP voters | 0.17 (0.45) |  |
| Abstainers | -0.00 (0.61) |  |
| **CPC undemocratic prime** |  |  |
| NDP voters |  | -0.25 (0.45) |
| Abstainers |  | -0.34 (0.58) |
| Observations | 215 | 219 |
| Controls | Yes | Yes |

Note: Entries are the average marginal effect of exposure to a party's undemocratic prime. Standard errors are shown in parentheses. The dependent variable has a scale ranging from 0-10, where 0 means 'undemocratic' and 10 means 'democratic'. Controls (age, gender, province, education, education, and income) are omitted from the table.

^+^ *p* < 0.10, ^*^ *p* < 0.05, ^**^ *p* < 0.01, ^***^ *p* < 0.001

### Table C.4. Moderating effect of attitude towards the convoy (LPC voters and CPC voters)

|  | (1) Placement of LPC by CPC voters | (2) Placement of CPC by LPC voters |
| --- | --- | --- |
| **LPC undemocratic prime** |  |  |
| Strongly agree | -2.37^*^ (1.01) |  |
| Somewhat agree | -1.03 (0.92) |  |
| Somewhat disagree | -0.71 (0.95) |  |
| Strongly disagree | 0.91 (0.85) |  |
| **CPC undemocratic prime** |  |  |
| Strongly agree |  | -0.09 (1.99) |
| Somewhat agree |  | -0.23 (1.06) |
| Somewhat disagree |  | 0.75 (0.95) |
| Strongly disagree |  | -1.20^*^ (0.50) |
| Observations | 194 | 201 |
| Controls | Yes | Yes |

Note: Entries are the average marginal effect of exposure to a party's undemocratic prime. Standard errors are shown in parentheses. The dependent variable has a scale ranging from 0-10, where 0 means 'undemocratic' and 10 means 'democratic'. The moderator variable has a four-point ordinal scale, which asks respondents how much they agree 'the protesters were protecting Canadians' rights and freedoms'. Controls (age, gender, province, education, education, and income) are omitted from the table.

^+^ *p* < 0.10, ^*^ *p* < 0.05, ^**^ *p* < 0.01, ^***^ *p* < 0.001

### Figure C.2. Recoding the moderator to binary (LPC voters and CPC voters)


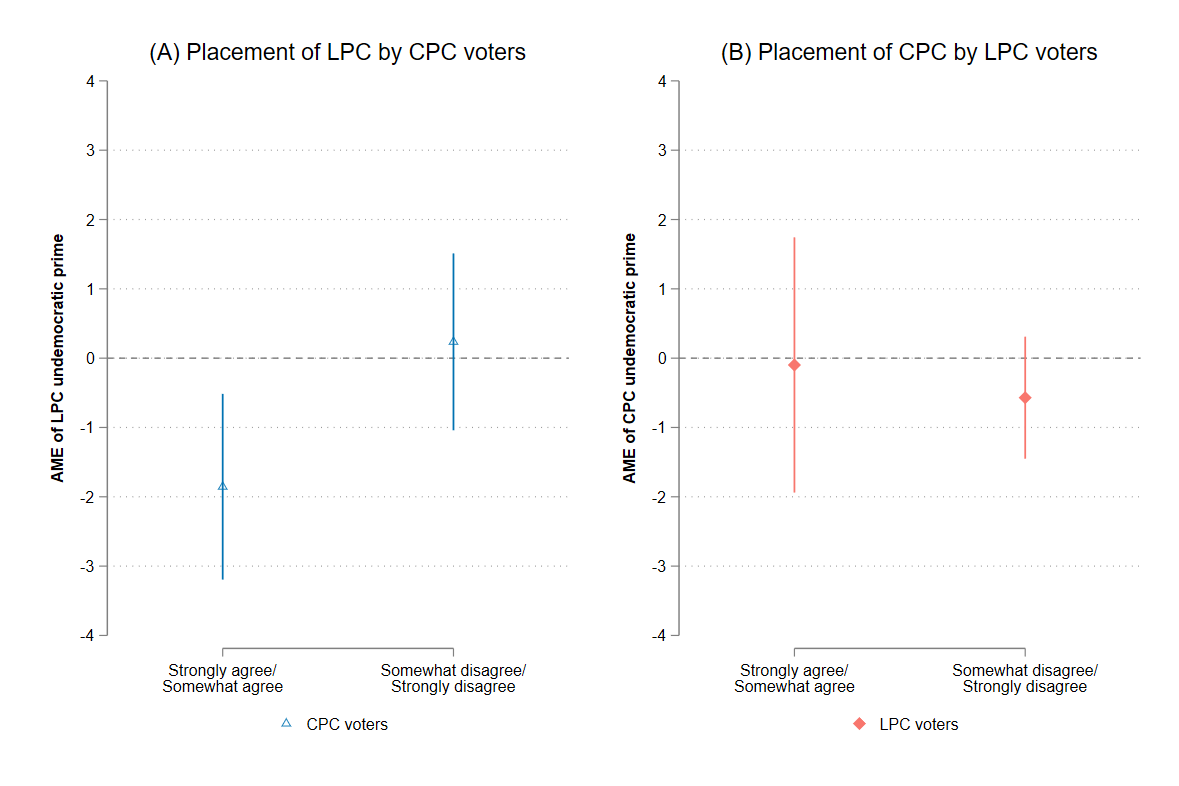


Note: The triangle and diamond indicate the average marginal effect of exposure to an undemocratic prime versus being in the control group. The dependent variable has a scale ranging from 0-10, where 0 means ‘undemocratic’ and 10 means ‘democratic’. In panels A and B, the moderator variable has a binary scale: “Strongly agree” and “Somewhat agree” are coded as 0, whereas “Somewhat disagree” and “Strongly disagree” are coded as 1. The question asks respondents how much they agree “the protesters were protecting Canadians’ rights and freedoms”. Spikes show 95% confidence intervals.

### Table C.5. Recoding the moderator to binary (LPC voters and CPC voters)

|  | (1) Placement of LPC by CPC voters | (2) Placement of CPC by LPC voters |
| --- | --- | --- |
| **LPC undemocratic prime** |  |  |
| Strongly agree/Somewhat agree | -1.85^**^ (0.68) |  |
| Somewhat disagree/Strongly disagree | 0.24 (0.65) |  |
| **CPC undemocratic prime** |  |  |
| Strongly agree/Somewhat agree |  | -0.10 (0.93) |
| Somewhat disagree/Strongly disagree |  | -0.57 (0.45) |
| Observations | 194 | 201 |
| Controls | Yes | Yes |

Note: Entries are the average marginal effect of exposure to a party's undemocratic prime. Standard errors are shown in parentheses. The dependent variable has a scale ranging from 0-10, where 0 means 'undemocratic' and 10 means 'democratic'. the moderator variable has a binary scale: 'Strongly agree' and 'Somewhat agree' are coded as 0, whereas 'Somewhat disagree' and 'Strongly disagree' are coded as 1. The question asks respondents how much they agree 'the protesters were protecting Canadians' rights and freedoms'. Controls (age, gender, province, education, education, and income) are omitted from the table.

^+^ *p* < 0.10, ^*^ *p* < 0.05, ^**^ *p* < 0.01, ^***^ *p* < 0.001

### Figure C.3. Moderating effect of attitude towards the convoy (NDP voters and Abstainers)


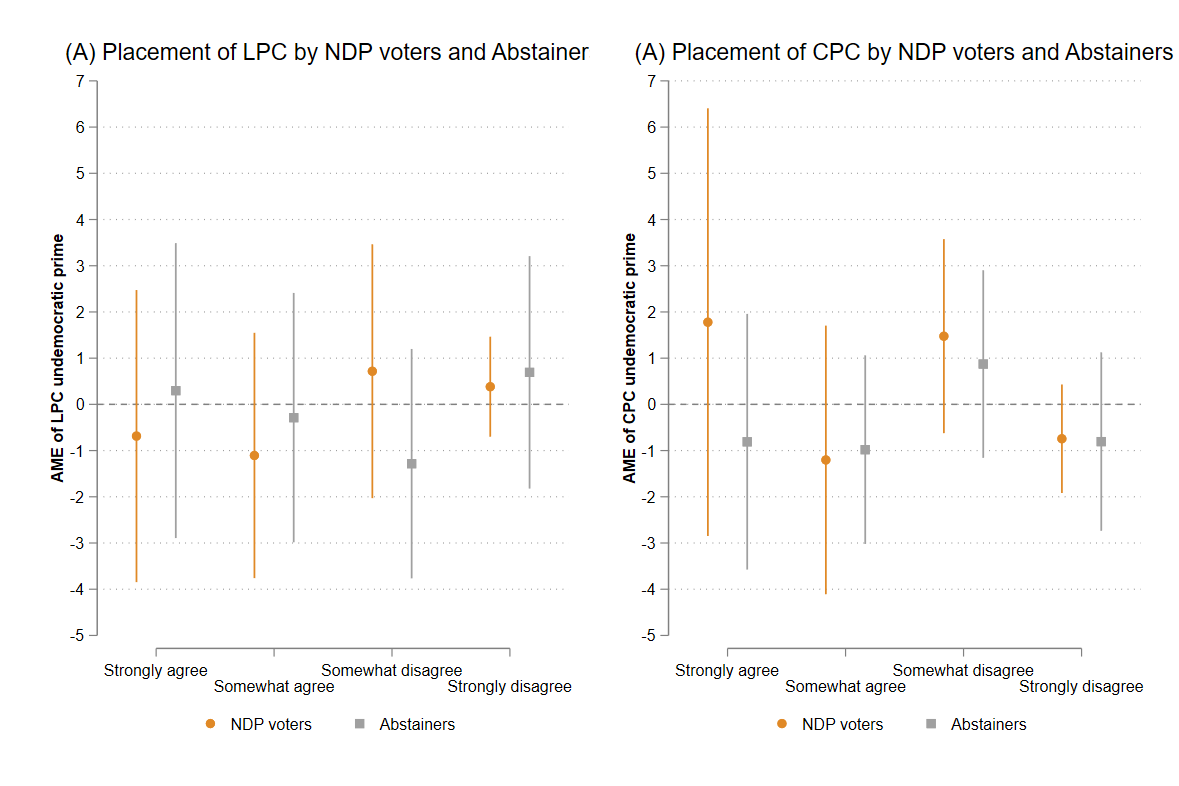


Note: The dot and square indicate the average marginal effect of exposure to an undemocratic prime versus being in the control group. The dependent variable has a scale ranging from 0-10, where 0 means ‘undemocratic’ and 10 means ‘democratic’. In panels A and B, the moderator variable has a four-point ordinal scale, which asks respondents how much they agree “the protesters were protecting Canadians’ rights and freedoms”. Spikes show 95% confidence intervals.

### Table C.6. Moderating effect of attitude towards the convoy (NDP voters and Abstainers)

|  | (1) Placement of LPC by NDP voters | (2) Placement of LPC by Abstainers | (3) Placement of CPC by NDP voters | (4) Placement of CPC by Abstainers |
| --- | --- | --- | --- | --- |
| **LPC undemocratic prime** |  |  |  |  |
| Strongly agree | -0.69 (1.60) | 0.30 (1.59) |  |  |
| Somewhat agree | -1.11 (1.34) | -0.29 (1.34) |  |  |
| Somewhat disagree | 0.72 (1.39) | -1.28 (1.24) |  |  |
| Strongly disagree | 0.38 (0.55) | 0.69 (1.25) |  |  |
| **CPC undemocratic prime** |  |  |  |  |
| Strongly agree |  |  | 1.78 (2.34) | -0.81 (1.38) |
| Somewhat agree |  |  | -1.20 (1.47) | -0.98 (1.02) |
| Somewhat disagree |  |  | 1.48 (1.06) | 0.87 (1.01) |
| Strongly disagree |  |  | -0.74 (0.59) | -0.81 (0.96) |
| Observations | 140 | 72 | 140 | 78 |
| Controls | Yes | Yes | Yes | Yes |

Note: Entries are the average marginal effect of exposure to a party's undemocratic prime. Standard errors are shown in parentheses. The dependent variable has a scale ranging from 0-10, where 0 means 'undemocratic' and 10 means 'democratic'. The moderator variable has a four-point ordinal scale, which asks respondents how much they agree 'the protesters were protecting Canadians' rights and freedoms'. Controls (age, gender, province, education, education, and income) are omitted from the table.

^+^ *p* < 0.10, ^*^ *p* < 0.05, ^**^ *p* < 0.01, ^***^ *p* < 0.001
